# Research Progress on Mechanical Properties and Fatigue Failure of Harmonic Drive Flexspline

**DOI:** 10.3390/s26134204

**Published:** 2026-07-03

**Authors:** Xiao Lian, Jianhui Liu, Youtang Li, Wuqiang Li

**Affiliations:** 1 School of Mechanical and Electrical Engineering, Lanzhou University of Technology, Lanzhou 730050, China; lianx2021@163.com (X.L.); liujh@lut.edu.cn (J.L.); lut_lwq@126.com (W.L.); 2 College of Intelligent Manufacturing, Longdong University, Qingyang 745000, China

**Keywords:** harmonic drive, flexspline, mechanical properties, fatigue failure, lifetime

## Abstract

**Purpose**—The flexspline of a harmonic drive constitutes a thin-walled structure with discontinuous gear rim and cylinder configuration, where cyclic stresses induce stress concentration, followed by crack initiation, propagation, and ultimately fatigue failure. This paper reviews advancements in understanding its mechanical properties and fatigue failure mechanisms, aiming to establish a foundation for enhancing operational longevity and guiding future research. **Design/Methodology/Approach**—The study integrates meshing theory, tooth shape parameters, cylinder stress influencers, and assembly/meshing stress considerations. Theoretical analysis, finite element simulations, and experimental methods are employed to examine stress patterns and fatigue dynamics. Structural parameters and tooth profiles are systematically analyzed for their impact on stress distribution and fatigue life. **Findings**—Flexspline fatigue failure arises from tooth root stress concentration and cylinder bending stress accumulation. The double-circular-arc tooth profile boosts load capacity by 35% relative to the involute profile, yet demands high-precision machining to preserve meshing performance. Increasing cylinder length mitigates stress concentration but reduces torsional stiffness, while optimized root fillet radii can lower the stress concentration coefficient by 28%. Assembly interference and meshing contact stress accelerate crack initiation, as validated by transient dynamics simulations. Surface strengthening processes (e.g., shot peening) enhance fatigue life by up to 66% through residual compressive stress regulation. **Originality/Value**—This paper synthesizes multi-scale research on flexspline design, structural optimization, and fatigue mechanisms, proposing novel approaches such as “manufacturability-oriented optimization” and digital twin-driven monitoring. By linking dynamic loads, material properties, and geometric parameters, it bridges theoretical gaps and provides actionable insights for high-precision harmonic drives in robotics and aerospace, advancing reliability in precision transmission systems.

## 1. Introduction

**Figure 1:**
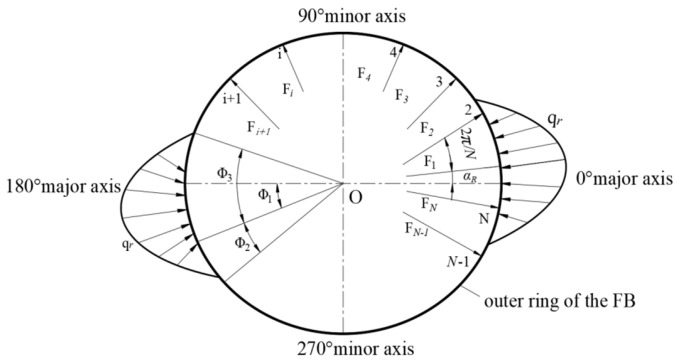
Force diagram of flexspline driven by wave generator.

With the continuous advancement of manufacturing technologies, the global industrial robotics sector has experienced rapid growth, with major manufacturing powers—including China, Japan, Germany, South Korea, and the United States—actively promoting the deployment of intelligent manufacturing systems. China’s industrial robotics sector has developed particularly rapidly, solidifying its position as the world’s largest robotics market. Data from China’s National Bureau of Statistics indicate that industrial robot production in 2025 reached 773,000 units, generating an estimated sales revenue of US$19.3 billion at an average industry price of US$25,000 per unit. In the January–May period of 2026, output stood at about 424,000 units, an increase of 28.1% year-on-year. The full-year production is forecast to hit 800,000 units, corresponding to a projected revenue of US$20 billion. Alongside this growth, harmonic drives—serving as high-precision transmission components—have attracted extensive research interest in major industrial nations. In Japan, Harmonic Drive Systems Inc. has long dominated the commercial market and contributed foundational work on tooth profile optimization [1,2]. In Europe, research groups in Germany and Italy have advanced the understanding of flexspline stress analysis and fatigue modelling [3,4,5]. In the United States, NASA and affiliated institutions have investigated harmonic drive performance under extreme operating conditions for aerospace applications [6,7,8]. Notably, as depicted in Figure 1, the harmonic reducer finds extensive application at the front-end joints of robots, contributing to over one-third of the manufacturing cost [7]. Given its high cost and critical role, the fatigue life of the flexspline directly determines the reducer’s overall reliability. However, during operation, the thin-walled structure of the flexspline leads to cyclical deformation, transitioning between long and short axes at every contact point under the influence of the wave generator. Torque transmission occurs through engagement with the circular spline at the long axis, rendering the toothed ring of the flexible wheel susceptible to localized stress concentration, thereby increasing the propensity for crack initiation and fatigue fracture, consequently diminishing the operational lifespan of the harmonic driver [8]. Given the imperative of ensuring robot reliability, reducing maintenance costs, and enhancing the fatigue resistance and longevity of the flexspline, research attention has gravitated towards investigating the stress and fatigue performance of this critical component.

**Figure 2:**
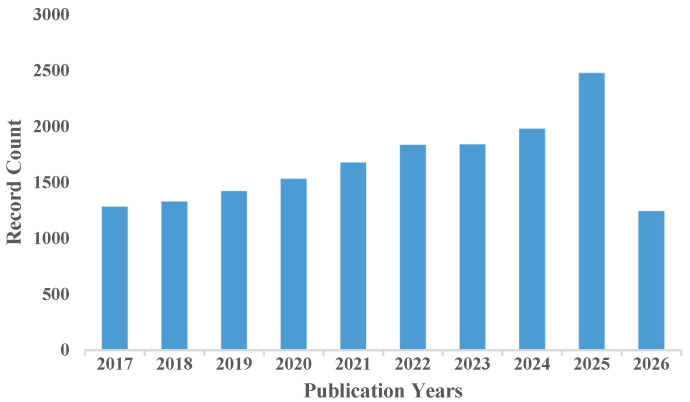
The number of publications on harmonic drive flexibles in the last 10 years.

**Figure 3:**
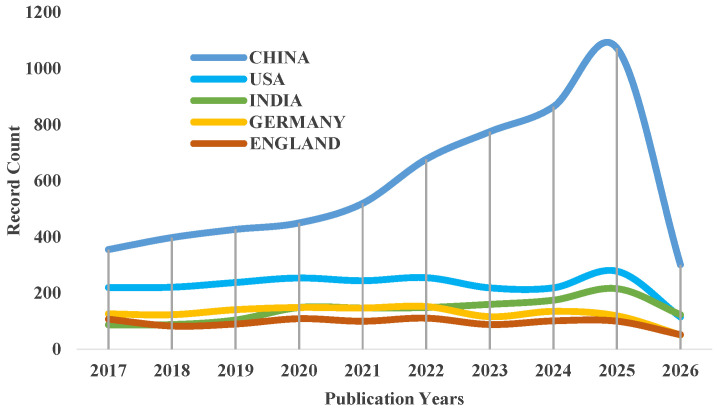
The curve of variation in the number of papers published by individual countries.

The harmonic drive technology has been flourishing around the world since the first harmonic drive worldwide was manufactured by Prof. C. Walton Musser in 1953 [9]. This paper focuses on the core issue that a harmonic drive should incorporate a flexspline. To identify relevant publications, a systematic literature search was conducted in the Web of Science Core Collection. The search was performed using the topic field (TS) with the exact keyword phrase “harmonic drive” to capture publications whose titles, abstracts, or author keywords contained this term. The search timeframe was set from January 2017 to 2026. The retrieved results were refined by document type, including only articles, dissertation theses, and review articles; other document types such as editorials, book reviews, and news items were excluded. This search strategy yielded a total of 15,279 publications for further bibliometric analysis. As shown in Figure 2, among the 16,628 publications, the number of papers is ranked as China (35.12%), the United States (13.6%) and India (8.4%), respectively, followed by Japan, South Korea, and Germany. The research focus varies by region: Chinese researchers have made substantial contributions to meshing theory, tooth profile design, and fatigue life prediction under multi-axis loading [10,11,12,13,14]; U.S.-based groups have advanced reliability analysis methods and accelerated life testing techniques [15,16,17]; Japanese researchers have contributed pioneering work on manufacturing precision and material selection for commercial flexsplines [18,19]; and European efforts have emphasized analytical and numerical modelling of flexspline deformation and stress [20,21,22,23]. Although the beginning of harmonic drive technology research in China was relatively late, the recent growth in research output from China, as shown in Figure 3, has significantly enriched the global knowledge base in this field. Thus, it is of great theoretical and practical significance to consolidate and critically examine the accumulated knowledge on the fatigue performance of harmonic drive flexsplines, with the aim of providing a reference for further advancing the design and reliability of harmonic drives across different application domains worldwide.

Drawing upon research conducted both domestically and internationally over the past decade, this paper provides an overview of the current state of investigation into the mechanical properties and fatigue failure of harmonic drive flexspline. This includes examinations into tooth geometry based on meshing theory, analyses of tooth parameters, evaluations of factors impacting cylinder stress, assessments of assembly and meshing-induced stresses on the flexspline, investigations into structural parameters influencing flexspline fatigue failure, explorations of testing methodologies, and discussions of fatigue theory. Building on this foundation, the paper also addresses ongoing research challenges regarding flexure stress and fatigue failure and outlines future development prospects.

## 2. The Mechanical Properties of the Harmonic Drive Flexspline

**Figure 4:**
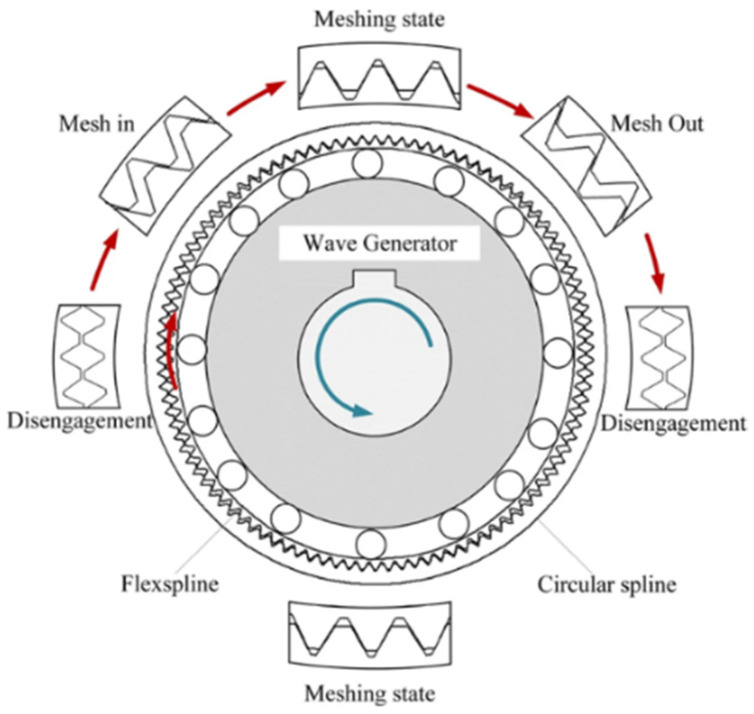
Harmonic drive process—diagram.

The thin barrel of the flexspline experiences continuous elastic deformation during rotation of the wave generator. As shown in Figure 4, this results in the inner surface of the gear rim making contact with the wave generator, while the outer teeth engage with the inner teeth of the circular spline during meshing. Additionally, the bottom of the barrel remains fixed and functions as an output member, subjecting the flexspline to complex forces [24], primarily including circumferential bending stresses, radial force reaction stress, bending stresses caused by the discrete distribution of the wave generator ball, and additional bending stresses at the tooth root during meshing. The operational efficacy of the harmonic gear transmission heavily relies on the overall stress experienced by the flexspline. Thus, stress analysis of the flexspline is pivotal in designing the service life of the harmonic driver. Factors influencing the stress distribution of the flexspline encompass the teeth configuration, design parameters, cylinder shape and structural parameters, and assembly stress.

Material selection is a core determinant of flexspline fatigue performance and service life. Currently, three categories of materials are widely adopted in commercial flexsplines:

Medium-carbon low-alloy steels (represented by 40CrNiMoA, 35CrMnSiA) [10]. After quenching and tempering, they feature high strength, good toughness and moderate cost, making them the most widely used materials for industrial robot flexsplines. Their fatigue failure is mainly dominated by non-metallic inclusion-induced crack initiation and tooth root bending fatigue.

### 2.1. The Stresses on Flexspline Gear Teeth of the Harmonic Drive

The tooth configuration of the flexspline stands as a pivotal factor influencing its lifespan, primarily impacting stress distribution and load transfer. Subsequently, these factors affect the fatigue life of the flexspline. During operation, the flexspline experiences compression from the wave generator, leading to passive deformation. This deformation alters the neutral layer curve of the flexspline and causes deformation in both the thin-walled cylinder and the gear teeth. Consequently, the theoretically designed mesh area decreases, leading to localized stress escalation, heightened wear, decreased precision, and diminished fatigue life.

#### 2.1.1. Overview of the Tooth Profile of the Harmonic Drive Flexspline

**Figure 5:**
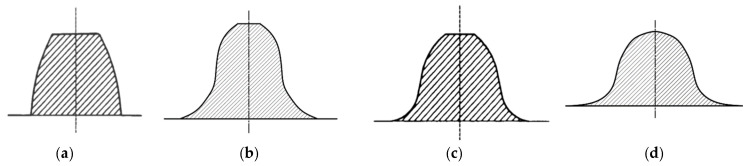
The tooth shape of the harmonic driver. (**a**) Involute tooth profile. (**b**) “S” tooth profile. (**c**) Double-arc tooth profile. (**d**) “P” tooth profile.

As illustrated in Figure 5, the current harmonic drive tooth shapes mainly include linear tooth shape, involute tooth shape, “S” tooth shape, circular arc tooth shape, and “P” tooth shape [25]. The linear tooth shape, the earliest form utilized in harmonic drivers, while adequate for meeting transmission ratio requirements, only accounts for radial deformation, thus neglecting tangential displacement and normal angle considerations necessary for high-performance harmonic transmission. Although the involute tooth form is well-established, it constitutes incomplete conjugate motion in harmonic transmission, leading to issues such as reduced overlap, heightened tooth load, and cusp meshing. These factors contribute to increased stress concentration at the root of the flexspline tooth, consequently diminishing the harmonic driver’s service life [26]. The “S” tooth shape, developed by Japan in the 1990s, offers superior meshing characteristics and an increased load rating compared to the involute tooth shape. However, it remains uncertain whether this tooth shape is theoretically ideal for harmonic gears. In contrast, the arc tooth profile, pioneered by Russian experts in the 1970s, features a large radius of curvature during meshing, enhancing contact strength. Despite widening the tooth groove and improving stress distribution at the root of the tooth, drawbacks include the non-strict conjugate relationship of the meshing tooth profile and a reduction in the meshing area between the circular spline and flexspline, resulting in manufacturing and processing challenges. The “P” tooth shape, independently developed by China Green, features low tooth height, thicker tooth width, increased meshing interval, and a larger arc radius at the root, minimizing flexure deformation and enhancing load-carrying capacity and anti-fatigue performance. However, this shape compromises the transmission accuracy of the harmonic driver. In Table 1, we summarize the advantages, disadvantages, and key performance characteristics of the five tooth profile types. Given the advantages of the double-arc tooth shape and its broad applicability, this paper predominantly focuses on reviewing research about this tooth shape.

#### 2.1.2. Progress in Research on Conjugate Tooth Shape in Harmonic Drives

The shape and size of teeth not only influence the motion performance and transmission efficiency of gears but also determine the contact space of the tooth surface during power transmission, known as the conjugate space. In gear transmission, ensuring full contact between the tooth surfaces of two gears is essential for enhancing load-carrying capacity and minimizing issues such as point-off, side-off, backlash, and other problems. Therefore, the matching of the gear tooth shape and the corresponding conjugate space holds particular significance.

(a) The investigation of conjugate tooth shape based on typical meshing theory

Methods commonly employed to derive the conjugate tooth shape in harmonic transmission based on meshing theory encompass the instantaneous centroid method, envelope method, improved kinematics method, and computer simulation, with the latter often used in conjunction with other techniques. The instantaneous centroid method intricately delineates gear geometry, facilitating analysis of transmission errors and tooth contact problems during gear operation. Simultaneously, the envelope method amalgamates deformation and curvature information of gear tooth profiles into an envelope curve, aiding in the evaluation of gear performance and vibration characteristics. Furthermore, the improved kinematics method enables analysis of contact stress and deformation in gear transmission, offering researchers a comprehensive understanding of kinematic behaviour and optimizing gear design to enhance transmission efficiency.

Dong et al. [27] introduced a tooth design approach employing the instantaneous centroid method, employing conjugate constraints at three specific points to concurrently design the double-arc tooth shape of the circular spline and flexspline, ensuring continuous, unbroken conjugate intervals in harmonic drive gear meshing. Drawing upon envelope theory and conjugate theory, Wang et al. [28] developed conjugate equations for the gear teeth in harmonic gearing, drawing upon the principles of envelope theory and improved kinematics-based conjugate theory. Through this endeavour, both exact and approximate conjugate theories for harmonic gearing were derived. Computational analysis indicates that the application of envelope mesh theory and harmonic mesh theory, rooted in enhanced kinematics, has minimal impact on the conjugate area and tooth contour of double-circular-arc tooth profiles within harmonic gearing. He X. et al. [29] investigated the severe stress concentration issue induced by large cone angles during the assembly and loading of short flexspline, which leads to diminished meshing contact areas. By employing neutral layer theory and envelope conjugation theories, the researchers proposed a double-circular-arc common-tangent tooth profile (DCTP) design combined with a longitudinal modification method for circular spline teeth, accompanied by the development of a comprehensive mathematical model. Their findings demonstrate that longitudinal tooth modification can effectively reduce the maximum contact pressure in harmonic drive systems by 69.6% while substantially enhancing the contact area of short flexspline. Wang et al. [30] designed the double-arc tooth profile for harmonic transmission flexible and circular spline based on the improved kinematic meshing theory of harmonic gear transmission, revealing that torsional stiffness could be enhanced by altering the meshing point during the meshing process.

(b) Study of conjugate tooth shape based on a neutral curve

The neutral curve of the harmonic drive flexspline is pivotal in elucidating the deformation characteristics of the flexspline, delineating the curve’s neutral line shape in the primary direction of change. The design of the flexspline hinges upon this neutral line. However, post-assembly, the flexspline undergoes deformation due to the wave generator’s influence, causing the corresponding deformation of the neutral circle. The extent and pattern of this deformation significantly impact the tooth configuration of the flexspline, its meshing state, and the torque transmission’s deformation. Notably, substantial changes correspond to heightened stress concentration, yielding elevated stress levels, while comparatively gradual changes relate to a more even stress distribution. Consequently, the neutral curve’s configuration not only influences the flexure’s overall rigidity and deformation but also shapes its stress distribution.

Zhen et al. [31] utilized a curve parallel to the cam shape curve to represent the neutral curve of the flexspline. They introduced a rotational transformation method for generating a more precise conjugate tooth profile, along with a direct generation method that bypasses the need for pre-designed tooth profiles. This rotational transformation approach achieves a superior conjugate tooth profile compared to the envelope method. Harmonic drivers designed through the direct generation method exhibit increased meshing teeth and depth, resulting in enhanced transmission accuracy and meshing force distribution compared to involute harmonic drivers. Notably, they possess higher transmission accuracy and meshing force distribution than involute harmonic drivers, and the increased meshing depth contributes to a higher load-carrying capacity.

Tang et al. [32] employed the cam profile curve as the driving parameter for the conjugate tooth profile, employing a curve mapping method to derive the convex tooth profile of the flexspline and the circular spline. Through geometric motion models and tooth profile conjugation conditions, they resolved the concave tooth profile of both wheels, proposing a harmonic transmission tooth profile design method driven by conjugate parameters capable of quadratic and two-point conjugation simultaneously. Zhu et al. [33] addressed the deformation of the cup body of the flexspline to formulate the spatial angles resulting from radial and tangential displacements. These angles accurately delineate the complex spatial motion relationship between the circular spline and the flexspline, thus significantly enhancing the load-carrying capacity and transmission performance of the harmonic driver. Song et al. [34] utilized the curve mapping method to resolve the tooth apex arc and employed the bidirectional conjugate method to address the tooth root arc of the harmonic driver, facilitating multi-tooth meshing and achieving greater meshing depth. To provide a concise overview of the above-mentioned approaches, Table 2 summarizes the four methods—instantaneous centroid method, envelope method, improved kinematics method, and neutral curve approach—in terms of their advantages, disadvantages, complexity, and representative applications.

**Figure 6:**
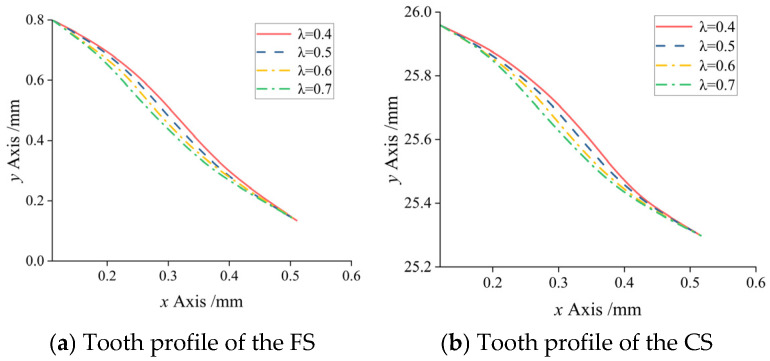
The influence of scaling coefficient λ on the tooth profile of harmonic gear.

**Figure 7:**
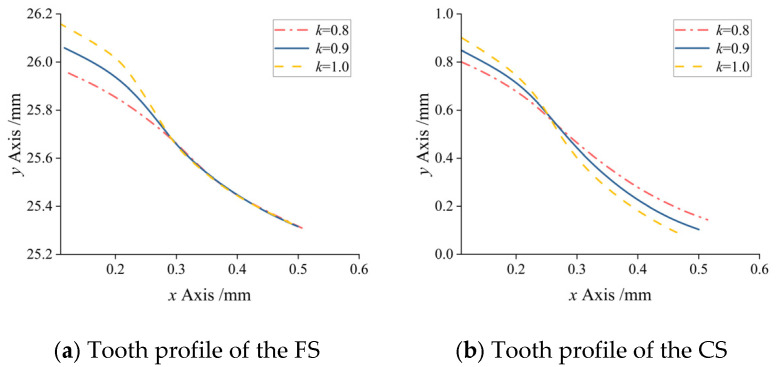
The influence of scaling coefficient *k* on the tooth profile of harmonic gear.

The critical parameters affecting the tooth profile of the harmonic drive—namely, the scale factor *λ* and the bending coefficient *k* of the flexspline—are analyzed and presented in Figure 6 and Figure 7. As shown in Figure 6, as λ decreases, the tooth thickness of the flexspline increases, causing its tooth profile to protrude outward, whereas the tooth thickness of the circular spline decreases with *λ*, resulting in a concave inward profile. Figure 7 illustrates the influence of the bending coefficient k on the tooth profiles of both the flexspline and the circular spline. The maximum deformation and the total tooth height of both components vary with k; specifically, tooth height increases with increasing *k*, although the pressure angle decreases correspondingly. A smaller bending coefficient *k* reduces the maximum deformation of the flexspline, which is beneficial for mitigating von Mises stresses during assembly and loading.

#### 2.1.3. Impact of Toothing Parameters on Flexspline Stresses

**Figure 8:**
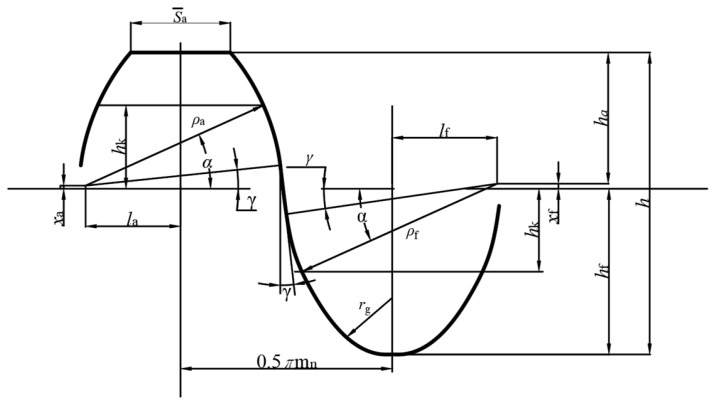
The schematic diagram of the double-circular-arc common-tangent tooth profile.

As illustrated in Figure 8, geometric parameters of the double-circular-arc tooth profile directly determine the meshing contact state and stress distribution of the flexspline. Reasonable parameter matching is a core approach to alleviate tooth root stress concentration and optimize the overall stress state. Hence, judicious selection of tooth profile parameters can mitigate stress concentration phenomena during gear meshing, consequently alleviating flexspline stress.

**Figure 9:**
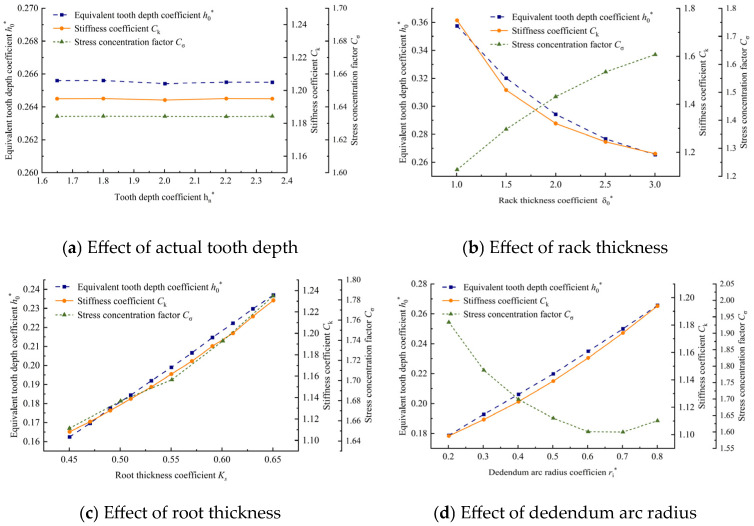
The variation in stiffness coefficient and stress concentration factor with geometric parameters.

The dedendum fillet radius is the most sensitive parameter for tooth root stress concentration. Mechanically, the tooth root is a typical geometric discontinuous structure, and bending stress from meshing loads will form a significant stress concentration effect at the sharp corner of the tooth root. Increasing the fillet radius can smooth the sudden geometric change and disperse the concentrated bending stress, thus reducing the stress concentration coefficient [35]. Figure 9 demonstrates the fluctuation of stress concentration coefficient with the tooth root arc radius, whereas the stiffness coefficient varies linearly with these parameters. Jiang et al. [36] devised a joint tooth profile devoid of tangent double circular arcs using the envelope method and conducted multi-body contact finite element analysis. The results indicated flexspline stress decreases and then increases with increasing root radius and root height coefficient, while other tooth shape parameters exhibit negative correlation with flexspline stresses. Zheng et al. [37] conducted failure analysis on a harmonic drive operating for 500 h and analyzed stress distribution in critical parts of the flexspline using LS-DYNA. They identified maximum stress around the working surface of the tooth root, attributing localized microcracks and variations in dimensional accuracy as primary causes for flexspline failure. Additionally, as the corner radius increases, the maximum stress in the cylinder wall of the flexspline exhibits a pattern of initially decreasing and then gradually increasing. This study offers valuable insights for the ongoing enhancement of flexspline design.

In summary, current research on the tooth profile of harmonic driver predominantly focuses on circular arc tooth profiles. While circular arc tooth profiles offer favourable meshing characteristics, existing studies primarily provide theoretical approximations of conjugate tooth profiles. Whether rooted in typical meshing theory or exploration of the neutral curve, these endeavours aim to find a suitable profile curve that accurately approximates the theoretical conjugate tooth shape. Concurrently, optimizing flexspline tooth profile parameters ensures proper meshing depth, top arc curve, root curve, and tooth thickness. This optimization leads to a larger conjugate interval, ensuring the occurrence of the “double conjugate” phenomenon in double-arc tooth profile harmonic transmission. This phenomenon significantly enhances the smoothness and load-carrying capacity of harmonic transmission.

### 2.2. Stress Analysis of the Flexspline Cylinder in the Harmonic Drive

The flexure barrel of a harmonic driver exhibits thinness and significant radial deformation, constituting a geometric nonlinear problem. Notably, the lifespan of the flexspline determines the overall longevity of the harmonic driver, underscoring the pivotal role of stress and lifespan considerations in its design. Bending deformation of the flexspline induces bending stress within its cylinder, primarily concentrated at the root and gradually diminishing across its width. Furthermore, a certain clearance exists between the flexspline and the gear, resulting in shear stress transfer from the gears to the flexspline cylinder under load. Consequently, investigating flexspline cylinder stress aids in optimizing the reliability and durability of the flexure cylinder, thereby enhancing the service life of the harmonic driver.

#### 2.2.1. Influence of the Structural Parameters on Cylinder Stresses

**Figure 10:**
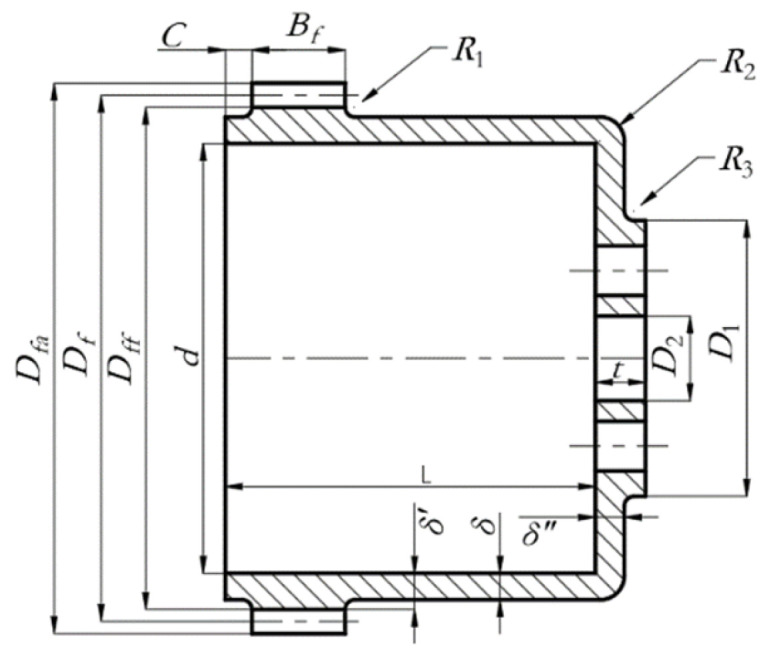
The structure parameters of flexspline.

The structural parameters of the flexspline, depicted in Figure 10, encompass the respective diameters, length, and wall thickness of the flexspline cylinder, the width of its toothed ring, and the radii of the transition round corners. These parameters play a direct role in influencing stress distribution and the load-carrying capacity of the flexspline.

Li et al. [38] developed a finite element model to investigate wave generator—flexspline contact mechanics, analyzing the mechanical characteristics of thin-walled flexspline and flexible bearings. The findings reveal a significant decrease in the maximum equivalent force of the flexspline with increasing cylinder length, while the thickness of the flexspline cylinder wall primarily influences force at its bottom. However, the impact of the maximum equivalent force value of the toothed ring and smooth cylinder is less evident. Similarly, the increasing trend of the maximum equivalent force value of the gear rim and smooth cylinder is not apparent. Ye et al. [39] introduced a method for assembly and meshing analysis of a circular spline—flexspline system based on rigid, flexible coupling theory and transient dynamics analysis. They observed higher stress in the flexspline during harmonic deceleration, with parameters such as tooth thickness, cylinder length, and width exerting the most significant impact on flexspline stress. Zuo et al. [40] utilized finite element software to conduct structural design and analysis of the flexspline in the B3-80 general purpose harmonic driver. Their findings demonstrate that as the cylinder length increases, the maximum equivalent force value of the flexspline decreases, resulting in a significant extension of service life. Consequently, augmenting the cylinder length can enhance the force condition of the flexspline, albeit at the expense of increased volume and decreased torsional stiffness. Furthermore, they observed an increase in the maximum equivalent force value of the flexspline body with increasing smooth cylindrical wall thickness, although the increasing trend is not pronounced. Zhang et al. [41], utilizing the response surface method and the central composite design (CCD) sampling method, conducted finite element analysis to ascertain factors influencing the stress of the flexspline. Their investigation revealed that radial deformation and radius exerted the greatest influence on stress, followed by the length-to-diameter ratio and thickness-to-diameter ratio. Increasing the thickness-to-diameter ratio can alleviate stress concentration at the back end of the toothed ring, ensuring optimal meshing and load-bearing capacity. In another study [42], the stiffness of the flexspline and stress at three observation points—inner wall, gear rim, and cup bottom—were analyzed. The results indicated that enlarging each chamfer structure of the flexspline effectively reduces stress concentration and enhances wheel stiffness. Zhu et al. [43] established a contact mechanics model for the flexure parts of a harmonic driver, analyzing the impact of flexspline cylinder length and bottom thickness on stress concentration. Increasing cylinder length improves flexspline stress in the harmonic driver, while bottom cylinder wall thickness primarily affects stress at the flexspline bottom, with minimal impact on the gear rim and smooth cylindrical cylinder stress. Wang et al. [44] proposed a rapid calculation method based on cylinder mechanical analysis and an equivalent additional bending moment model. Their findings demonstrate a proportional relationship between circumferential stress and cylinder length, inversely proportional to the square of the flexible part’s length. The method’s accuracy was verified through finite element simulation.

Based on the above studies, Table 3 summarizes the key structural parameters of the flexspline—including cylinder length, wall thickness, diameter, chamfer radius, cup bottom thickness, and thickness-to-diameter ratio—along with their respective effects on stress distribution. This classification provides a comprehensive overview of the most influential geometric variables reported in the literature.

#### 2.2.2. Analysis of Stress in Different Parts of the Cylinder

During operation, the inner ring of the gear’s front end in a harmonic drive makes contact with the wave generator, experiencing deformation due to its action. Stress distribution varies across components such as the meshing teeth, toothed ring, cylinder, bottom of the cylinder, and the flange at its base, leading to different locations for crack generation and destruction. Therefore, studying stress distribution within the cylinder is imperative.

Chen et al. [45] examined the span value (M-value) deviation of the flexspline, circular spline M-value deviation, and long half-axis deviation of the wave generator. Their analysis revealed that maximum stress on the flexspline occurs at the engagement zone of the flexspline tooth surface under the joint action of the circular spline and cam. Stress on the flexspline tooth increases with the elongation of the cam’s long half-axis or the flexspline’s M-value but decreases with an increase in the circular spline’s M-value. Sahoo et al. [46] employed finite element techniques to investigate stress distribution in the flexspline cup, identifying maximum bending stress at the bottom flange of the cylinder. Moreover, they observed an increase in average stress with applied torque. Yi et al. [47] conducted simulations and analyses to investigate the intricate stress–strain distribution of the cup-shaped harmonic driver. Through experimental verification of the assembly and loading processes, their study elucidates that, subsequent to the assembly of the wave generator and the flexspline, the maximal stress on the flexspline concentrates at the root position of the tooth ring’s rear end. Additionally, the presence of the circular spline induces an escalation in stress levels for both the flexspline and the bearings. Under load, the torque application leads to a characteristic folding-up trend in the stress increase observed on the flexspline.

Based on the current research, it is evident that key structural parameters of the flexspline, such as cylinder length, diameter, wall thickness, and transition fillet, exert significant influence on its stress levels. Increasing cylinder length diminishes bending stress and interference fit stress concentration, thereby reducing maximum equivalent stress and extending fatigue life. However, it should be noted that elongating the cylinder results in increased volume and decreased torsional stiffness. Maintaining a reasonable thickness-to-diameter ratio for the flexspline cylinder wall promotes stress reduction, but exceeding an optimal thickness leads to elevated maximum stress levels. The wall thickness of the flexspline cup bottom has a pronounced impact on force distribution, whereas its effect on maximum stress in the gear rim and smooth cylinder is less apparent. Enlarging each chamfer structure of the flexspline effectively mitigates stress concentration and enhances flexspline stiffness. Notably, stresses are higher in the gear rim section and the flange bottom of the flexspline cylinder due to engagement and fixed connection output, making these areas susceptible to fatigue damage.

### 2.3. Impact of Assembly and Meshing on Flexspline Stresses

During the assembly process of the harmonic driver, various components must be assembled, leading to deformation and stress in the flexspline. Additionally, assembly forces or pressure may be applied to the flexspline, inducing additional stresses that could impact its overall stress and lifespan. Moreover, during normal operation of the harmonic driver, meshing gears generate pressure and moments on the flexspline, potentially causing stress concentration areas and fatigue damage. Thus, investigating the influence of assembly and meshing on the stress of the flexspline is imperative.

Mahanto et al. [48] investigated stress–strain characteristics in the cup body of the flexspline under different assembly configurations, including unassembled circular spline, assembled conventional cam, and split cam. They observed that while the overall stress in the cup body of the flexspline was higher with the split cam, the strain at the contact between flexspline teeth and circular spline teeth during engagement was reduced, thus lowering the likelihood of tooth failure. Chen et al. [49] examined the impact of wheel teeth on the assembly stress of the flexspline gear rim. They found that the bending stiffness increases coefficient and stress concentration coefficient varied with changes in ring wall thickness and groove width. Moreover, they noted that the flexspline could withstand alternating stress twice as much as assembly stress, with circumferential stress from bending moment contributing the highest proportion to assembly stress. Deng et al. [50] conducted contact analysis and experimental verification of the assembly process of the flexspline in a hollow harmonic driver. They found that radial deformation was predominant under the action of the wave generator, with stress concentration occurring at the connection between the gear rim corresponding to the long axis of the wave generator and the barrel, as well as at the root of the tooth at the end of the flexspline teeth. Deng [51] enhanced the design of the flexure wheel and formulated a model for the rigid–flexure wheel system. Subsequently, mechanical properties and fatigue life of the flexure wheel were simulated and analyzed concerning assembly and meshing. The findings reveal elevated stress levels in the flexure wheel during both assembly and meshing phases. It is evident that a comprehensive analysis of harmonic gears necessitates thorough consideration of these two processes to enhance the rationality and accuracy of analytical outcomes. Sahoo et al. [52] verified the stress distribution in the harmonic driver at full load through experimental and finite element analysis of bending stresses in the flexspline cups, and investigated the stress distributions at the primary and secondary contact points at full load condition, proving the existence of secondary tooth contacts in the involute harmonic gearing and their contact strengths are proportional to each other. For the harmonic gearing mechanism in the transmission process flexspline subjected to alternating loads and cyclic deformation, Zhang et al. [53] analyzed stress distribution of assembled flexspline under no-load conditions, finding stress magnitude and distribution correlated with the wave generator, with highest stress at gear rim and decreasing stress along the cup’s axial direction. Pacana J et al. [3] numerically calculated the stresses in a harmonic drive flexure ring and investigated the effect of the type of wave generator on the stress distribution of the flexspline and found that high stresses existed in the deformed region of the flexspline as well as in the edge of the ring, and that the maximum stress values were consistent with the long and short axes of the flexspline in each cross-section. The stress distribution of the flexspline under load and no-load conditions under the action of different forms of wave generators is verified through experiments, and it is obtained that the cam wave generator can ensure the optimal meshing of the teeth of the flexspline and the circular spline.

In summary, the interference assembly between the long axis of the wave generator and the flexspline leads to stress concentration or local stress changes, with the gear rim experiencing the highest stress while stress on the cylinder gradually decreases along the axial direction. Additionally, assembly-induced flexspline deformation results in circumferential stress from bending moments, comprising a significant portion of assembly stress. Moreover, during meshing, the contact between flexspline and circular spline teeth yields the highest stress at the contact point, proportionate to the contact strength. Both the assembly and engagement phases significantly influence the stress level of the flexspline, necessitating thorough consideration of both processes in stress analysis.

## 3. Current Research on Fatigue Failure of Flexspline

The flexspline, as the pivotal component of the harmonic drive, facilitates power and motion transmission through controlled elastic deformation during operation, making it susceptible to fatigue damage due to cyclic alternating stresses over extended periods. Consequently, fatigue damage to the flexspline constitutes a common failure mode in harmonic gearing structures, impacting the operational integrity of the harmonic drive. Moreover, the inability to compress the flexspline to a smaller size due to overall structural strength constraints impedes harmonic drive development. Therefore, investigating flexspline reliability holds paramount importance for enhancing meshing performance and extending the harmonic drive’s lifespan.

### 3.1. Impact of Structural Parameters on Flexspline Fatigue Performance

As mentioned, whether long or short cylinder types, key structural parameters including cylinder length, wall thickness, transition fillet, tooth thickness, and tooth width all influence cylinder stress. It is evident that greater deformation and stress correlate with increased susceptibility to fatigue failure, thereby affecting the flexspline’s fatigue life.

**Figure 11:**
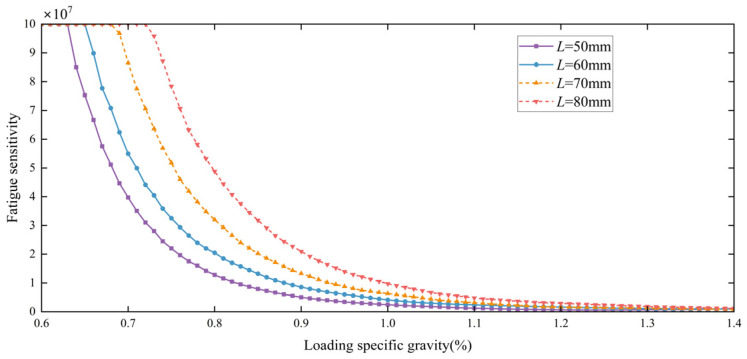
Effect of loading specific gravity on the fatigue characteristics of flexsplines with different cylinder lengths.

**Figure 12:**
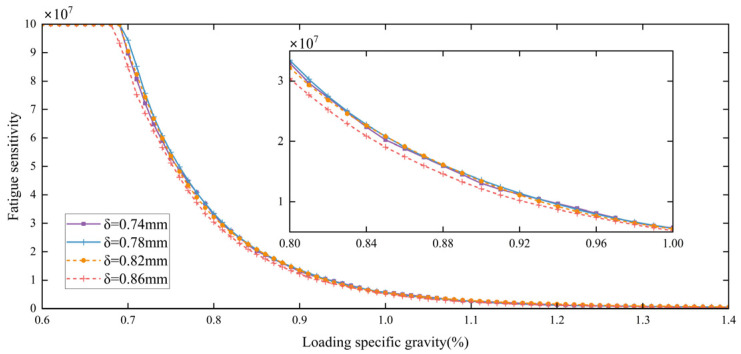
Effect of loading specific gravity on fatigue characteristics of flexsplines with different wall thickness.

Ye et al. [39] investigated the influence of flexspline structure and material parameters on fatigue life sensitivity, highlighting the significant impact of tooth thickness, tooth width, and cup length. Consequently, these structural parameters should be carefully managed within a reasonable range during flexspline design to enhance the design life of harmonic gears. Yang et al. [40] designed the B3-80 universal harmonic gear drive flexspline, analyzing the impact of cylinder length and wall thickness on flexspline fatigue life via FEM. Figure 11. illustrates that as load weight increases from 60% to 140% while maintaining constant cylinder length (L), the fatigue-sensitive characteristic curve of the flexspline becomes flatter. Similarly, Figure 12. demonstrates that as load weight increases within the same range while keeping cylinder wall thickness constant, the fatigue sensitivity curve of the flexspline gradually becomes less steep. Conversely, increasing cylinder wall thickness minimally affects the fatigue sensitivity curve of the flexspline, with variations in wall thickness at the cylinder bottom showing insignificant impact on fatigue life. Cheng et al. [54] established a mathematical model to calculate flexspline fatigue strength and analyzed the sensitivity of each structural parameter to fatigue strength coefficient. They found that among these parameters, maximum radial deformation of the flexspline exhibits the highest sensitivity, followed by flexspline wall thickness, radius, and least sensitivity observed in cylinder length. Yu [55] analyzed flexspline fatigue life using smooth cylindrical shell theory and ANSYS software, revealing that length and tooth width significantly affect stress and fatigue life, with parameters ranked in descending order of influence: length, tooth width, transition angle of tooth root, gear rim thickness, and flexspline wall thickness. Yang et al. [56] examined XB1-60 harmonic gear drive flexspline stress via finite element analysis, observing a stage-wise upward trend in the effect of cylinder length on fatigue life and high nonlinearity in the effect of wall thickness, with significant stress concentration improvement within the 0.4–0.5 mm wall thickness range. Moreover, fatigue life sharply decreases within 5–8 mm tooth width range and changes smoothly between 8 and 12 mm. Consequently, gear tooth width selection should align with specific load-bearing requirements, prioritizing smaller values to enhance flexspline service life.

In conclusion, the flexspline’s structural parameters exhibit a sensitive zone, wherein variation within a reasonable range reduces stress, thereby enhancing design life; conversely, stress increases and lifetime decreases. For instance, increased cylinder length disperses load, reducing stress concentration and improving flexspline fatigue life, yet excessively long cylinders may compromise torsional stiffness. Similarly, wider tooth width distributes load more evenly, slowing surface fatigue damage, but within the sensitive zone, fatigue life decreases sharply. Moreover, larger root circle radii alleviate stress concentration at tooth bases, while increased cylinder wall thickness enhances flexspline stiffness, reducing deformation and stress concentration. Additionally, larger cylinder diameters decrease stress concentration and deformation, contributing to improved fatigue life within a certain range. In a reasonable design range, the parameters influencing the fatigue life of the flexspline, ranked in descending order of significance, are as follows: length, tooth width, tooth root transition fillet, ring thickness, and flexspline wall thickness.

It should be noted that there are certain differences in the quantitative conclusions of parameter sensitivity among different studies, which are mainly caused by the differences in research assumptions, object specifications and evaluation methods. First, the boundary conditions of numerical models are inconsistent: some studies only consider the meshing load of gear teeth, while others introduce assembly interference, flexible bearing support stiffness and dynamic load factors, resulting in deviations in stress distribution calculation and sensitivity ranking. Second, the research objects have different structural forms and size specifications: for conventional long cup-type flexsplines, cylinder length is the most sensitive parameter affecting fatigue life; while for short cup-type or hat-type flexsplines with limited axial size, the influence of wall thickness and bottom transition fillet is more significant, and the sensitivity ranking will change accordingly. Third, the material systems and surface states adopted in the studies are different: for medium-carbon alloy steel flexsplines dominated by bending fatigue, the sensitivity of tooth root fillet is higher; for high-strength maraging steel flexsplines, the influence of structural parameters on fatigue life is relatively weakened due to the high toughness of the material. In addition, different evaluation dimensions (stress concentration coefficient, theoretical fatigue life, experimental failure cycle) will also lead to differences in the quantitative amplitude of parameter influence. At present, the mainstream sensitivity ranking conclusion is mainly applicable to conventional medium-sized cup-shaped harmonic drive flexsplines made of alloy structural steel. For flexsplines with special structures, special sizes or special materials, the influence law of parameters needs to be further verified through targeted research. The differences in existing studies also reflect that the current research has not yet formed a unified general quantitative evaluation system for the fatigue performance of flexsplines with different specifications, which is also an important direction to be broken through in future research.

### 3.2. Experimental Study of Flexspline Fatigue Life

Experimental research offers a quantitative assessment of harmonic drive flexspline fatigue life. This investigation can encompass fatigue tests, dynamic load tests, accelerated life tests, acoustic emission tests, multi-factor optimization tests, and numerical simulation predictions.

Zheng et al. [37] conducted a failure analysis of a harmonic drive under 500 h of operation, identifying localized microcracks on the gear surface as the primary cause of flexspline failure. These microcracks led to abnormal contact and localized rupture, resulting in diminished dimensional accuracy and reduced service life. To enhance life assessment accuracy, Wang et al. [57] proposed an accelerated life test programme for harmonic drives, considering flexspline fatigue fracture and overall machine transmission error as failure criteria. Zhang et al. [58] developed a dedicated accelerated life test (ALT) model for harmonic drives in space systems, employing physical statistics and Mason’s fatigue damage rule to describe the fatigue failure process. Their findings support the practicality of the cumulative damage approach in estimating harmonic drive life in space mechanisms. Zhang et al. [59] utilized the shot peening process to modify the residual stress on the surface of the flexspline, thereby enhancing its fatigue resistance. The study demonstrated a significant improvement in the flexspline ‘s fatigue resistance, with an observed increase in fatigue life by 66%. It was noted that the impact of residual stress on fatigue life is more pronounced when surface roughness is minimal, while residual stress becomes predominant under higher levels of surface roughness. Additionally, an increase in surface roughness was found to correspond to a fluctuating trend in fatigue life. In addition to shot peening, other surface treatment processes have also been explored to enhance the fatigue performance of flexspline and precision gear components [60]. Nitriding forms a hardened diffusion layer that effectively suppresses surface fatigue crack initiation. DLC coating features an ultra-low friction coefficient of 0.05–0.15 and a high hardness of 15–80 GPa, which significantly improves wear resistance. Compound surface modification, i.e., shot peening followed by DLC coating, integrates the compressive residual stress field from shot peening with the low-friction and high-hardness surface from DLC coating. For gear steel, the fatigue life after shot peening, DLC coating, and compound modification increased by 3.68, 2.35, and 3.36 times, respectively, compared to untreated specimens [61]. Shot peening remains the most cost-effective solution for fatigue-dominated industrial applications. DLC coating is more suitable for lubrication-limited or space environments, though its adhesion to the substrate remains a critical concern. Nitriding offers balanced improvements in both wear and fatigue resistance, but its high processing temperature of 500–600 °C may cause thermal deformation in thin-walled flexsplines. Compound modification provides the most comprehensive protection when both fatigue and wear are critical, but at the expense of higher cost and process complexity [62]. Zhao et al. [63] investigated and delineated the fatigue failure mechanism of four domestically manufactured flexspline components. Their analysis identified the principal culprit behind premature fatigue fractures: the substandard purity of the parts. This deficiency leads to the presence of large-sized Al_2_O_3_ brittle inclusions and elongated MnS strips within the flexspline material. Notably, these inclusions and strips, extending through the plate thickness direction, serve as critical sites for fracture initiation. León D et al. [64] estimated contact and bending stresses of the harmonic driver using nonlinear dynamics and finite element (FE) analysis. They determined that the contact stress on the flexspline teeth is less than the bending stress at the tooth root, suggesting that pitting poses minimal concern for the harmonic driver. Given that bending fatigue is significantly influenced by design parameters, an appropriate design can achieve a safety factor exceeding 2.5. Shu et al. [65] conducted fatigue analysis on the transition angle of the flexure wheel’s barrel bottom flange. By developing harmonic driver finite element software and establishing an experimental platform, they identified shear stress at the corner of the barrel bottom flange as the primary cause of fatigue fractures. Natsuda et al. [66] utilized finite element analysis to investigate fatigue failure in the flexspline, pinpointing the long axis of the flexspline gear rim as the primary site of fatigue failure. Additionally, due to fixed constraints at the bottom of the flexspline barrel, the barrel bottom flange emerged as the weakest point susceptible to fatigue failure and reduced fatigue life.

Primarily, flexspline fatigue cracks dominate the damage of the harmonic drive. Fatigue testing serves to characterize and analyze fatigue fractures, elucidating crack generation and expansion mechanisms in fatigue failure. This aids in the quest for suitable surface modification technologies to alter residual stress on the flexspline surface, thereby enhancing its fatigue life. However, due to the diverse sizes and shapes of flexsplines, different size-sensitive zones exist, necessitating time-consuming experimental studies. Hence, experimental studies of flexspline often opt for accelerated life tests or combine finite element analysis with experimental studies to expedite the testing cycle.

### 3.3. Theoretical Study of Flexspline Fatigue Life

The fatigue life of the flexspline is influenced by various factors such as the torsional stiffness of components, assembly clearances, manufacturing accuracy, and load distribution between teeth. Theoretical calculations of fatigue strength are complex, with assumptions in formula derivation differing from actual conditions [55]. Therefore, practical applications should consider these factors and establish reasonable modelling and assumptions to ensure reliable research methods and results. Theoretical research on flexspline fatigue life includes stress analysis and modelling, fatigue performance assessment, fatigue life prediction models, cycle counting, fatigue life assessment based on prediction models, and cycle numbers. Fatigue theory-based life models employ various methods including damage mechanism-based models, empirical formula-based models, finite element analysis-based models, damage accumulation-based models, and stress intensity factor-based models.

Yan et al. [67] obtained the load spectrum of the harmonic drive using the Miner linear cumulative damage criterion as shown in Equation (1). They observed that the maximum fatigue damage value occurs at the end and root of the flexspline tooth, indicating initial fatigue damage at these locations. Subsequently, damage progresses along the axial line of the flexspline from inside the tooth to the cup bottom, with more severe damage at the cup mouth compared to the inner wall of the flexspline in contact with the wave generator.

(1) $$D=\sum_{i=1}^{l}n_{i}/N_{i}$$

The total damage *D* is calculated using the Miner linear cumulative damage rule, where *N_i_* represents the number of fatigue cycles corresponding to different stress amplitudes (i.e., fatigue life), and *n_i_* denotes the number of load cycles for various stress amplitudes. Zhang et al. [17] introduced a time-varying reliability analysis method that accounts for material fatigue damage deterioration, fatigue limit, and load uncertainty, as shown in Equation (2).

(2) R(n)=p(g(X,n)>0)=1−p(g(X,n)<0)=1−F(−β(n))

where *R*(*n*) denotes the time-dependent reliability of the harmonic drive; g(X,n) is the time-dependent state function of the harmonic drive, with *X* being the vector of random variables; β(n) is the time-dependent reliability index; and *F*( ) denotes the cumulative distribution function of the standardized state function. Under the common assumption that the state function is normally distributed after normalization, *F*( ) reduces to the standard normal CDF *Φ*( ), and Equation (2) yields R(n)=1−F(−β(n))=1−Φ(−β(n))=Φ(β(n)). They assessed the time-varying reliability of the harmonic drive under different loads by integrating the residual strength model, stochastic uptake method, and Edgeworth level. Their findings indicate a concordance between simulation and test results. Moreover, fatigue fracture of the flexspline is localized at the root of the tooth behind the gear rim.

Cai et al. [68] employed mathematical and simulation methods to examine the stress and fatigue life of the multi-toothed root in a harmonic drive. They compared the safety coefficients of various materials using the S-N curve fatigue curve method, as depicted in Table 4. Fatigue life was assessed using the Goodman and Gerber fatigue theories, where *S* represents the fatigue safety coefficient of the flexspline, *σ*_(−1)_ denotes the ultimate fatigue stress, *σ_b_* is the material’s strength limit, *σ_m_* signifies the average stress, and *σ_a_* indicates the stress amplitude. 40CrNiMoA, as the most widely used benchmark material for commercial industrial flexsplines, was set as the control group in this study. The findings reveal that the fatigue life safety coefficient of 40CrNiMoA exceeds that of other materials by a minimum of 12.95%. This comparison offers valuable insights for evaluating the fatigue life of the flexspline and selecting suitable materials. Feng et al. [69] developed an active learning Kriging model to predict the fatigue life of a flexure wheel, accounting for uncertainties in material properties and load conditions. They observed a threefold difference between the maximum and minimum predicted life values, highlighting the significant influence of random uncertainties in material and load on flexure wheel fatigue life. Wang et al. [70] integrated theoretical calculations with finite element analysis to assess the fatigue life of the flexure wheel. They noted a rapid decrease in the fatigue coefficient with increasing load, with fatigue rupture becoming likely when the theoretical load exceeded by approximately 10%. These research findings offer theoretical and technical insights into flexible wheel structure design, stress analysis, and fatigue optimization.

In summary, establishing a fatigue life calculation model involves utilizing the damage criterion and stress–life curve of the material, along with flexspline characteristics. Theoretical calculations determine the fatigue damage value, identifying vulnerable areas and crack propagation paths. However, this process relies on experimental data, necessitating their integration with experimental methods or simulations. It is worth noting that while these methods can assess flexspline fatigue life, linear theory-based approaches may be inadequate due to the geometric nonlinearity arising from significant flexspline deformations.

### 3.4. Semi-Quantitative Parameter Sensitivity Analysis

Previous studies have qualitatively investigated the influence of flexspline structural parameters on fatigue performance, but lack a unified sensitivity classification and priority ranking for the main design parameters. Based on the numerical trends, simulation curves and research conclusions from the existing literature, a literature-driven qualitative sensitivity evaluation is adopted in this section.

According to the amplitude of stress and fatigue life variation, the steepness of performance changing curves and the degree of influence stated in published studies, five core structural parameters are classified into four sensitivity levels: extremely sensitive, highly sensitive, moderately sensitive and weakly sensitive.

To clarify the influence law and design guidance of each parameter, the qualitative sensitivity classification of flexspline structural parameters is summarized in Table 5.

Combined with literature analysis, the sensitivity order of the five core parameters from high to low is concluded as:

cylinder length > tooth width > tooth root transition fillet > ring thickness > flexspline wall thickness.

In practical design, extremely sensitive and highly sensitive parameters should be taken as the key optimization objects to suppress stress concentration and improve fatigue life. Moderately sensitive and weakly sensitive parameters can be reasonably selected on the premise of satisfying meshing performance, structural stiffness and manufacturing process, so as to balance the reliability, compactness and economy of harmonic drive flexsplines.

## 4. Conclusions and Future Perspectives

### 4.1. Summary of Core Quantitative Conclusions

This study systematically reviews the research progress on mechanical properties and fatigue failure mechanisms of harmonic drive flexsplines. By sorting out numerical results from published theoretical, simulation and experimental studies, the core quantitative conclusions are summarized as follows:

(1) Tooth profile and meshing performance

The double-circular-arc common-tangent tooth profile combined with longitudinal modification of circular spline teeth can reduce the maximum contact pressure in harmonic drives by 69.6% and significantly expand the contact area of short flexsplines.

Compared with the traditional involute tooth profile, the double-circular-arc tooth profile achieves a larger conjugate meshing interval and higher load-carrying capacity, with improved meshing smoothness.

(2) Structural parameter sensitivity

The sensitivity of core structural parameters to flexspline fatigue life is ranked from high to low: cylinder length > tooth width > tooth root transition fillet > ring thickness > flexspline wall thickness.

Cylinder length and tooth width are extremely sensitive parameters: small fluctuations in the two parameters will cause sharp changes in stress and fatigue life, with obvious sensitive intervals.

Optimizing the tooth root fillet radius can effectively reduce the stress concentration coefficient; within the range of 0.4–0.5 mm wall thickness, the stress concentration of the flexspline is significantly improved.

(3) Fatigue performance improvement by surface treatment

Shot peening treatment introduces residual compressive stress on the flexspline surface, which can increase the fatigue life of the flexspline by up to 66%.

For the widely used medium-carbon low-alloy steel 40CrNiMoA, its fatigue safety coefficient is at least 12.95% higher than that of other common alloy structural steels under the same stress condition.

### 4.2. Future Research Directions and Challenges

Although significant progress has been made in the research on stress characteristics and fatigue life of flexsplines, there are still key challenges to be solved due to the structural complexity of flexsplines and the diversity of working conditions. Future research can be carried out in the following directions:

(1) Manufacturability-oriented complex tooth profile design

As concluded in Section 2.1, current tooth profile optimization mostly focuses on theoretical meshing performance, and the coordination between design accuracy and machinability is insufficient. Future research can combine additive manufacturing and ultra-precision machining technology with topology optimization and machine learning algorithms to realize the transition from theoretical optimal design to manufacturable optimal design of high-performance tooth profiles such as double-circular-arc profiles.

(2) Digital twin-driven full-cycle fatigue monitoring

As summarized in Section 2.3 and Section 3.2, the dynamic stress data of flexsplines under actual working conditions is insufficient, and there is a lack of high-precision life prediction methods combined with real-time states. In the future, embedded sensing technology and Internet of Things technology can be deployed to establish a real-time stress monitoring system for key regions. Combined with digital twin multi-physics field coupling models, the dynamic evolution of fatigue damage can be simulated to achieve high-precision life prediction.

(3) Multi-parameter coupling fatigue failure mechanism

As discussed in Section 2.2 and Section 3.1, existing studies mostly analyze the influence of single parameters, and the coupling mechanism of structural parameters, material properties and external loads on fatigue failure is not clear. Future research can adopt multi-objective optimization and uncertainty quantification methods, combined with multi-body dynamics simulation and in situ experiments, to establish a hybrid load spectrum database and clarify the failure threshold and safety margin under extreme working conditions.

(4) Cross-scale fatigue research combining microstructure and macroscopic performance

As analyzed in Section 3.3, the micro-mechanism of fatigue crack initiation in flexspline materials is still insufficient. Future research can adopt a micro-meso-macro multi-scale perspective to study the influence of material microstructures such as grain boundaries and inclusions on crack initiation. Meanwhile, new high-entropy alloy materials and multifunctional surface modification technologies such as diamond-like carbon coatings and self-healing coatings can be further developed to inhibit crack initiation from the surface and extend the service life of flexsplines.

With the growing demand for high-precision transmission in robotics, aerospace and other fields, improving the reliability of harmonic drive flexsplines will remain a core research priority. Future efforts should deepen industry–academia–research collaboration, follow the technical path of intelligence, green and high performance, and promote the continuous upgrading of high-end equipment manufacturing technology.

## Figures and Tables

**Table 1:** Comparison of advantages, disadvantages, and key performance characteristics of the five tooth profile types.

Tooth Profile	Advantages	Disadvantages	Key Performance
Linear	Simple; basic requirement.	Ignores tangential, normal; low capacity.	Low load, high stress.
Involute	Mature; well-known.	Incomplete conjugate; cusp meshing.	Moderate load, high stress.
“S”	Good meshing; higher rating.	Uncertainty; complex.	High load, moderate stress.
Circular Arc	High contact strength; good root stress.	Manufacturing difficulty; less meshing area.	High load, lower stress.
“P”	Best fatigue/load; low deformation.	Accuracy loss.	Highest load, lowest stress.

**Table 2:** Comparison of methods for conjugate tooth profile analysis in harmonic drives. “+” is Advantages; “−” is Disadvantages.

Method	Key Features (Advantages/Disadvantages/Complexity)	Typical Applications
Instantaneous centroid	+ Precise geometry description; analyzes errors & contact. − Limited to specific points; not fully continuous. Complexity: moderate.	Double-arc design (Dong et al. [27]); multi-point conjugation.
Envelope	+ Integrates deformation/curvature; provides exact/approx. theories. − Computationally intensive; needs accurate deformation models. Complexity: high.	Conjugate equations (Wang et al. [28]); double-arc analysis.
Improved kinematics	+ Analyzes stress/deformation; optimizes efficiency; enhances stiffness. − Complex kinematic modelling; less intuitive for large deformations. Complexity: moderate to high.	Stiffness optimization (Wang et al. [30]); contact analysis.
Neutral curve	+ Directly tied to real deformation; precise profile generation; enables multi-tooth meshing. − Highly dependent on curve model; stress distribution sensitive. Complexity: high.	Rotational transformation (Zhen et al. [31]); curve mapping (Tang et al. [32]); spatial angle method (Zhu et al. [13]); bidirectional conjugate (Song et al. [34]).

**Table 3:** Summary of key structural parameters affecting flexspline stress.

Parameter	Effect of Increasing Parameter	Key References
Cylinder length (L)	↓ Maximum equivalent stress; ↓ bending stress; ↑ fatigue life; but ↑ volume; ↓ torsional stiffness.	Li et al. [38]; Ye et al. [39]; Zuo et al. [40]; Zhu et al. [43]; Wang et al. [44]
Cylinder wall thickness (t)	↑ Stress at cylinder bottom (significant); minimal effect on gear rim & smooth cylinder.	Li et al. [38]; Zuo et al. [40]; Zhu et al. [43]
Cylinder diameter (D)	Affects stress via length-to-diameter ratio; radial deformation and radius have greatest influence on stress.	Zhang et al. [41]
Chamfer/fillet radius (r)	↓ Stress concentration; ↑ flexspline stiffness.	Zhang et al. [42]
Cup bottom thickness	↑ Stress at cylinder bottom; minimal effect on gear rim & smooth cylinder.	Zhu et al. [43]
Thickness-to-diameter ratio (t/D)	↓ Stress concentration at rear end of toothed ring; ensures optimal meshing and load-bearing capacity.	Zhang et al. [44]

Arrows indicate the direction of change: ↓ denotes a decrease, ↑ denotes an increase.

**Table 4:** Fatigue formulas and safety factor.

Fatigue Theories	Fatigue Formulas	Safety Factor (*S*)
Gerber	$\sigma_{a}=\sigma_{-1}\left[1-{\left(\frac{\sigma_{m}}{\sigma_{b}}\right)}^{2}\right]$	$\frac{S\sigma_{a}}{\sigma_{-1}}+{\left(\frac{S\sigma_{m}}{\sigma_{-1}}\right)}^{2}=1$
Goodman	$\sigma_{a}=\sigma_{-1}\left[1-\left(\frac{\sigma_{m}}{\sigma_{b}}\right)\right]$	$\frac{\sigma_{a}}{\sigma_{-1}}+\frac{\sigma_{m}}{\sigma_{b}}=\frac{1}{S}$

**Table 5:** Qualitative sensitivity classification of flexspline structural parameters.

Sensitivity Grade	Structural Parameters	Influence Characteristics
Extremely sensitive	Cylinder length, tooth width	Small parameter fluctuation induces sharp change in stress and fatigue life with obvious sensitive interval.
Highly sensitive	Tooth root transition fillet	Nonlinear influence with optimal interval for stress reduction; obvious improvement on meshing performance.
Moderately sensitive	Ring thickness	Gentle performance variation and limited regulation effect on fatigue life.
Weakly sensitive	Flexspline wall thickness	Limited effect on stress distribution and fatigue life.

## Data Availability

The data supporting the findings of this study are available from the corresponding author upon reasonable request.
